# Effects of the prophylactic use of escitalopram on the prognosis and the plasma copeptin level in patients with acute cerebral infarction

**DOI:** 10.1590/1414-431X20208930

**Published:** 2020-10-07

**Authors:** Jin-Xia Cao, Li Liu, Yun-Tao Sun, Qing-Hong Zeng, Yan Wang, Jie-Chun Chen

**Affiliations:** 1 Department of Neuropsychiatry, The Second People's Hospital of Lianyungang, Lianyungang, Jiangsu, China; 2 Department of Neurology, The Second People's Hospital of Lianyungang, Lianyungang, Jiangsu, China; 3 Department of Laboratory, The Second People's Hospital of Lianyungang, Lianyungang, Jiangsu, China

**Keywords:** Acute cerebral infarction, Escitalopram, Prognosis, Copeptin, Stroke

## Abstract

This study aimed to investigate whether the routine administration of escitalopram for three months would improve the prognosis of patients with ischemic stroke and decrease the plasma copeptin level. A total of 97 patients with acute cerebral infarction were randomly allocated to receive escitalopram (5-10 mg once per day, orally; n=49) or not to receive escitalopram (control group; n=48) for 12 weeks starting at 2-7 days after the onset of stroke. Both groups received conventional treatments, including physiotherapy and secondary prevention of stroke. The National Institutes of Health Stroke Scale (NIHSS) score was used to evaluate the disability of patients at the initial evaluation and at the monthly follow-up visits for three months. Impairment in the daily activities was assessed using the Barthel Index (BI), while cognitive impairment was assessed using Mini-Mental State Examination (MMSE) score. The psychiatric assessment included the administration of the Present State Examination modified to identify Diagnostic and Statistical Manual of Mental Disorders (DSM-IV) symptoms of depression. The severity of depression was measured using the 17-item Hamilton Rating Scale for Depression (HAMD). During the 3-month follow-up period, 95 patients were included in the analysis (two patients withdrew from the escitalopram group). NIHSS and BI improvement at the 90th day were significantly greater in the escitalopram group (P<0.05), while HAMD and plasma copeptin levels significantly decreased, compared to the control group (P<0.01). In patients with acute ischemic stroke, the earlier administration of escitalopram for three months may improve neurological functional prognosis and decrease copeptin level.

## Introduction

Cerebrovascular disease is the most common disease in the nervous system. There are approximately 2.5 million new-onset patients in China every year, and number of patients increases by 10% yearly. Ischemic stroke accounts for the vast majority (70-80%) (1-3). Even though thrombolytic therapy, intravascular therapy, and stroke unit care can be carried out in the acute stage of cerebral infarction, due to the time window or other limiting factors, 50-75% of patients continue to have different degrees of disability, which accounts for the first disability cause in adults. All these have seriously influenced the mental and physical well-beings and life quality of the stroke survivors and have caused tremendous medical and economic burden and social pressure. Hence, there is an urgent need to explore other treatment strategies to improve the prognosis (1-3).

Some clinical evidence has shown that the early prophylactic use of selective serotonin reuptake inhibitors (SSRIs) in patients with cerebral infarction can not only prevent the occurrence of post-stroke depression (PSD), but also promote neurological rehabilitation, especially motor function of patients without depression after cerebral infarction, and thereby improve the prognosis (4-7). Previous research has suggested that SSRIs might regulate the function of the hypothalamic-pituitary-adrenal (HPA)-axis in the treatment of depression (8). Arginine vasopressin (AVP) is a sensitive indicator that can reflect the activation level of the HPA-axis and correlate with the state and prognosis of cerebral infarction (9). AVP receptor antagonists can also play a significant role in cerebral protection (10). However, due to its short half-life, it is difficult to determine the content of AVP. Copeptin is the C-terminal part of AVP, which is released in the same amount as AVP. Since it is stable *in vivo* and can be easily detected, the level of copeptin can be an ideal substitute to reflect the level of AVP (11). It has been shown that the plasma copeptin level is correlated with the prognosis of acute ischemic stroke (12-14). Due to the great harm of cerebral infarction and limited treatment methods, a prospective randomized clinical case-control study was conducted to investigate the prognosis of the prophylactic use of the antidepressant escitalopram in acute cerebral infarction and its effect on the plasma copeptin levels in order to provide both a relevant theoretical basis and guidance for clinical treatment.

## Material and Methods

### General materials

All patients were hospitalized patients treated for acute ischemic stroke from January 2013 to April 2016. The inclusion criteria were patients with acute anterior circulation cerebral infarction for the first time that met the diagnostic criteria for the early diagnosis and treatment guidelines for medical and health professionals formulated by the American Heart Association/American Stroke Association in 2013 (15). All patients were hospitalized within one week after the onset and diagnosed by cranial magnetic resonance image (MRI). The exclusion criteria were as follows: 1) patients with recurrent stroke, hemorrhagic stroke, or posterior circulation stroke; 2) patients with PSD or previous history of depression; patients receiving mood stabilizers, antipsychotics, or any antidepressant before enrollment; 3) patients with depression caused by other organic brain diseases, or depression caused by psychoactive substances and non-addictive substances; 4) patients with consciousness disorder, aphasia, and dementia; 5) patients with a 17-item Hamilton Rating Scale for Depression (HAMD) of ≥17; patients with a National Institutes of Health Stroke Scale (NIHSS) score of ≥20; 6) patients with a previous history of cancer and psychosis; 7) patients with previous chronic obstructive pulmonary disease, heart failure, pulmonary, hepatic or renal failure, or other severe chronic diseases; 8) patients with serious suicidal tendencies, and the laboratory and accessory examinations revealing obvious coagulation dysfunction; and 9) patients who refused to participate or cooperated badly.

A total of 97 patients were included, and the random number table method was used to randomly allocate 49 patients into the treatment group and 48 patients into the control group.

### Treatment methods

Patients in the treatment group received routine treatments for cerebrovascular diseases, including secondary prevention of cerebral infarction, brain protection therapy, and rehabilitation. No antidepressant was allowed to be used and insomniacs, such as zolpidem or benzodiazepines could be administered for a short time. Patients in the treatment group received prophylactic escitalopram in addition to the basic therapies (trade name: escitalopram oxalate, produced by Xi'an Yangsen Pharmaceutical Co., Ltd., China; Tablets; Specifications: 10 mg, 5-10 mg/d, oral administration in the morning consecutively for three months).

### Follow-up and treatment evaluation

All patients were assessed before treatment and followed-up monthly after treatment. The depressive symptoms were assessed using the HAMD Scale. PSD was diagnosed according to the DSM-4 diagnostic criteria for post-stroke depression and with the HAMD Scale score >17. The incidence of PSD was registered. The NIHSS score was used to evaluate neurological deficits, the Barthel Index (BI) was applied to evaluate daily living activities, and the Mini-Mental State Examination (MMSE) was used to assess cognitive function. The treatment evaluation was conducted by the same physician, who was blinded to the patient's clinical data.

### Plasma copeptin concentration assay

Five milliliters of blood was drawn from the anterior elbow vein in the morning at fasting before and at three months after treatment. The samples were anticoagulated and centrifuged (2500 *g*, for 10 min at 4^o^C). The upper layer supernatant was placed in EP tubes, sealed, and stored in a refrigerator at -80°C. The plasma samples were tested in the same batch. ELISA double antibody sandwich method was used to determine the level of copeptin. The kit was provided by Shanghai Bogu Biotechnology Co., Ltd. (China) and the detection was performed by specially-assigned personnel who strictly followed manufacturer’s instructions.

### Adverse reaction and laboratory data detection

Routine blood test, urine test, and stool test were carried out before and after drug administration together with assays of hepatic and renal function, blood glucose, lipid profiles, electrolytes, and ECG. Any adverse reaction was recorded.

### Statistical analysis

SPSS 13.0 statistical software package (IBM, USA) was used for data processing. The data are reported as means±SD. The means of the quantitative data were compared by single factor analysis of variance (ANOVA) or non-parametric test. Chi-squared test was used for counting data analysis and repeated measurement ANOVA was used for comparison of variables before and after the measurement. P<0.05 was considered statistically significant.

## Results

### Basic characteristics of patients in each group

Forty-nine patients were allocated to the treatment group. Among them, 26 were males and 23 were females. They were 42-85 years old, with a mean age of 62±10.9 years. For education background of these patients, 15 patients finished primary school, 23 patients finished middle school, and 11 patients were university graduates. The lesions were located in the left frontal cortex and basal ganglia in 35 patients and in the right frontal cortex and basal ganglia in 14 patients.

The control group consisted of 48 patients: 22 males and 26 females. They were 45-82 years old, with a mean age of 63±9.7 years. For education background of these patients, 11 patients finished primary school, 24 patients finished middle school, and 13 patients were university graduates. The lesions were located in the left frontal cortex and basal ganglia in 31 patients and in the right frontal cortex and basal ganglia in 17 patients.

There were no significant differences in gender, age, education background, lesion location between the two groups (P>0.05), which suggested that the two groups were comparable.

### NIHSS, BI, MMSE, and HAMD at different time points during the follow-up between the two groups

There was no difference in NIHSS, BI, MMSE, and HAMD scores at enrollment between the two groups. At different time-points during the post-treatment follow-up, the NIHSS and HAMD scores were significantly lower in the escitalopram intervention group than in the control group (P<0.05 and P<0.01, respectively). Furthermore, the BI scores were significantly higher in the treatment group than in the control group (P<0.01), while the higher MMSE scores were not significantly different (P>0.05) (Table 1).

**Table 1:** Comparison of NIHSS, BI, MMSE, and HAMD at different time points during the follow-up of ischemic stroke patients treated or not with escitalopram.

Assessment	Treatment group (n=47)	Control group (n=48)	P
NIHSS			
Pre-treatment	8.16±1.04	8.38±1.12	0.34
1 month post-treatment	3.72±0.89	5.31±0.79	0.03
2 months post-treatment	2.47±0.94	4.31±1.07	0.02
3 months post-treatment	1.57±0.68	3.78±0.91	0.01
BI			
Pre-treatment	48.69±10.45	49.16±9.83	0.61
1 month post-treatment	72.31±9.83	56.84±9.47	0.01
2 months post-treatment	86.57±7.93	70.69±8.14	0.01
3 months post-treatment	93.73±6.74	76.83±7.26	0.01
MMSE			
Pre-treatment	22.35±3.72	22.78±4.31	0.78
1 month post-treatment	24.17±3.59	23.57±4.35	0.52
2 months post-treatment	25.49±3.86	24.32±3.89	0.27
3 months post-treatment	26.24±3.57	25.06±3.67	0.08
HAMD			
Pre-treatment	12.13±3.23	11.94±3.53	0.47
1 month post-treatment	7.92±1.86	14.63±4.34	0.01
2 months post-treatment	7.44±1.72	13.79±3.64	0.01
3 months post-treatment	5.27±1.84	14.72±4.15	0.01

Data are reported as means±SD. NIHSS: National Institutes of Health Stroke Scale; BI: Barthel Index; MMSE: Mini-Mental State Examination; HAMD: Hamilton Rating Scale. Repeated measurement ANOVA was used for comparisons.

### Incidence of PSD between the two groups post-treatment

PSD (incidence) in the escitalopram intervention group at 1, 2, and 3 months post-treatment were three cases (6.1%), two cases (4.1%), and zero case, respectively, with a total of five cases (10.2%). In the control group, the number of cases of PSD (incidence) were thirteen cases (27.1%), seventeen cases (35.4%), and twenty cases (41.7%), respectively at those time-points, with a total of twenty-five cases (50.3%). There was a significant difference between the two groups at each same period (P<0.05 and P<0.01, respectively).

### Plasma copeptin level between the two groups at post-treatment

There was no statistical difference in the copeptin level between the two groups before treatment (P>0.05), while in the post-treatment follow-up, the levels of copeptin in the escitalopram intervention group were significantly lower compared with the control group (P<0.05, Table 2).

**Table 2:** Comparison of plasma copeptin level of ischemic stroke patients treated or not with escitalopram at post-treatment.

Plasma copeptin level	Treatment group (n=47)	Control group (n=48)	P
Pre-treatment	14.84±1.42	13.92±2.01	0.43
3 months post-treatment	6.56±0.87	10.43±1.24	0.01

Data are reported as mean±SD in pmol/L. Repeated measurement ANOVA was used for comparison.

### Adverse reactions

In the escitalopram intervention group, 47 patients finished the 3-month treatment, while one patient discontinued treatment due to dizziness and another due to blurred vision. All 48 patients in the control group finished treatment. There was no obvious change in the blood, urine, and stool routine tests, hepatic and renal function, electrolytes, and ECG between the two groups three months after treatment. During treatment, two cases of dizziness, four cases of drowsiness, seven cases of dry mouth, and six cases of constipation occurred in the drug intervention group, and these symptoms disappeared after symptomatic treatment.

## Discussion

The results of the present study showed that the prognosis of the patients treated with escitalopram was better than that of patients in the control group. In the escitalopram treatment group, the NIHSS and HAMD scores significantly decreased, the incidence of PSD significantly decreased, the BI significantly increased, and the plasma copeptin levels significantly decreased, compared to patients treated with routine therapies for cerebral infarction, which is consistent with some previous research results (4-7). Cognitive impairment is a common and important complication of stroke, with the prevalence ranging widely from 10 to 82% (16). The MMSE score is a brief standardized instrument that has been extensively used to assess individuals' cognitive function. In this study, MMSE scores were increased in the escitalopram treatment group, but the difference compared to the control group was not statistically significant. The small sample size of this study may have contributed to this result.

Escitalopram, as one of the SSRI antidepressants, is presently the most widely used first-line drug for the treatment of anxiety, depression, and PSD. Its rapid onset, efficacy, safety, and tolerance have been widely verified. In recent years, some clinical studies have shown that the early use of SSRI antidepressants not only significantly reduces the incidence of PSD, which is independent of its antidepressant effects, but also promotes neuronal regeneration and plasticity changes, comprehensively improving the post-stroke motor, emotional regulation, cognitive, and social functions, together with the prognosis of stroke (4-7,17-19). In addition, some studies have shown that the earlier antidepressant drugs are used after ischemic stroke, the better the rehabilitation effect will be (17,18), while delayed administration might miss the best rehabilitation opportunity. In view of the serious outcome of cerebral infarction and its high incidence, high missed diagnosis rates, and the great risk of PSD, the early prophylactic use of SSRI antidepressants is particularly important for the prevention of PSD, especially for the rehabilitation of cerebral infarction.

The detailed mechanisms of SSRI antidepressants on the improvement of cerebral infarction prognosis remain unclear. Previous studies have shown that SSRI antidepressants may play a protective role in the brain through the anti-inflammatory mediators, improving blood supply to ischemic brain tissues, promoting the regeneration of hippocampal neurons, and regulating the functions of the autonomic nervous system and neuroendocrine system, so as to improve the prognosis of cerebral infarction and prevent and treat PSD (4-7). In the acute phase of cerebral infarction, the body is in a stress state activated by the HPA-axis. As a stimulating factor in the HPA-axis, AVP may reflect the degree of stress and aggravate the neuronal damage in patients with cerebral infarction and is positively correlated with the severity of infarction (9). However, AVP is very unstable in plasma. Most of the AVP binds to platelets and is cleared quickly, which limits its clinical application. A study conducted by Katan et al. (12) indicated that copeptin could better reflect individual stress level and the activation of the HPA axis compared with the pulsed-secretion of cortisol. Thus, copeptin has become an independent predictor for the prognosis of cerebral infarction (12-14). Furthermore, a previous study revealed that SSRI antidepressants might regulate the function of the HPA axis and are correlated with the therapeutic effects in the treatment of depression (8). Some recent studies have also indicated that escitalopram could significantly regulate the function of the HPA axis and the secretary rhythm of cortisol (20). In the present study, the results revealed that the levels of plasma copeptin in patients with cerebral infarction decreased, and the level in the escitalopram treatment group was significantly lower (P<0.05) than that in the control group after three months of treatment. All these suggest that escitalopram might promote the recovery of neurological function, improve the prognosis of patients with cerebral infarction, and prevent the occurrence of PSD by regulating the function of the HPA axis and reducing the level of copeptin. As far as we know, there is no report on the changes of copeptin levels before and after the prophylactic use of escitalopram after acute cerebral infarction.

Our study had several limitations. First, our sample size was small limiting the statistical power of the analyses. Second, the follow-up period in this study was comparatively short. Third, the MMSE used in this study is a screening tool for cognitive assessment. It has substantial restrictions because it does not measure the individual's whole cognitive ability. Other tools to assess the cognitive function were lacking.

In summary, the prophylactic use of escitalopram might provide significant therapeutic effects, which might be due to the regulation of the function of the HPA axis, decreasing the plasma copeptin level and promoting the recovery of neural function. As a result, escitalopram could reduce the degree of neurological deficit and depression, prevent PSD, improve the daily living ability of patients, and effectively improve the prognosis of patients with acute cerebral infarction. This treatment has few side effects and might be considered for clinical prophylactic application.
